# Systematic review and meta-analysis of the biomechanical properties of the human dura mater applicable in computational human head models

**DOI:** 10.1007/s10237-022-01566-5

**Published:** 2022-03-09

**Authors:** Quinton Pearcy, Joanna Tomlinson, Justyna A. Niestrawska, Dustin Möbius, Ming Zhang, Johann Zwirner

**Affiliations:** 1 Department of Anatomy, University of Otago, Dunedin, New Zealand; 2 Division of Macroscopic and Clinical Anatomy, Gottfried Schatz Research Center, Medical University of Graz, Graz, Austria; 3 Institute of Legal Medicine, University Medical Center Hamburg-Eppendorf, Hamburg, Germany; 4 Institute of Legal Medicine, University of Leipzig, Leipzig, Germany

**Keywords:** Biomechanical properties, Computational modeling, Dura mater, Elastic modulus

## Abstract

Accurate biomechanical properties of the human dura mater are required for computational models and to fabricate artificial substitutes for transplantation and surgical training purposes. Here, a systematic literature review was performed to summarize the biomechanical properties of the human dura mater that are reported in the literature. Furthermore, anthropometric data, information regarding the mechanically tested samples, and specifications with respect to the used mechanical testing setup were extracted. A meta-analysis was performed to obtain the pooled mean estimate for the elastic modulus, ultimate tensile strength, and strain at maximum force. A total of 17 studies were deemed eligible, which focused on human cranial and spinal dura mater in 13 and 4 cases, respectively. Pooled mean estimates for the elastic modulus (*n* = 448), the ultimate tensile strength (*n* = 448), and the strain at maximum force (*n* = 431) of 68.1 MPa, 7.3 MPa and 14.4% were observed for native cranial dura mater. Gaps in the literature related to the extracted data were identified and future directions for mechanical characterizations of human dura mater were formulated. The main conclusion is that the most commonly used elastic modulus value of 31.5 MPa for the simulation of the human cranial dura mater in computational head models is likely an underestimation and an oversimplification given the morphological diversity of the tissue in different brain regions. Based on the here provided meta-analysis, a stiffer linear elastic modulus of 68 MPa was observed instead. However, further experimental data are essential to confirm its validity.

## Introduction

With the abrupt rise of biomechanical research based on computational models (Chafi et al. 2009; Kleiven 2003; Viano et al. 2005; Zhang et al. 2001a) and the fabrication of synthetic tissue grafts that exhibit lifelike biomechanical characteristics (Kizmazoglu et al. 2019; Nunamaker et al. 2011), the need for high-quality biomechanical properties of human tissues has become increasingly evident. Overall, the biomechanical characterization of human tissues is still scarce. Several factors give rise to the lack of information available, including but not limited to the availability of fresh cadaveric tissues, the expertise of research teams in anatomy and engineering or financial challenges to buy required testing equipment. As a prime example, the biomechanical characterization of the dura mater was investigated further throughout the past three years (Aydin et al. 2019; Kizmazoglu et al. 2019; Zwirner et al. 2019a, b, c, 2020). The biomechanical properties of cranial dura mater are paramount to select and fabricate appropriate artificial substitutes for duraplasty, such as the Gore-Tex Expanded Cardiovascular Patch (W.L. Gore & Associates Inc., Flagstaff, AZ, USA), the Durepair graft (Medtronic Inc., Goleta, CA, USA), or the Tutopatch (Tutogen Medical GmbH, Neunkirchen am Brand, Germany) (Kizmazoglu et al. 2019). Furthermore, the biomechanical properties of the dura mater are required when it is used as a “model tissue” to answer fundamental research questions on the material behavior of collagen-rich tissues (Zwirner et al. 2019a, 2020). Moreover, the biomechanical properties of cranial dura mater are required to accurately simulate the tissue in computational head models to answer predominantly impact-related research questions (Chafi et al. 2009; Kizmazoglu et al. 2019; Viano et al. 2005; Zhang et al. 2001a). For the latter, an elastic modulus of 31.5 MPa that was observed in dynamic vibration tests 50 years ago (Galford and McElhaney 1970) is most frequently applied (Chafi et al. 2009; Kleiven 2003; Viano et al. 2005; Zhang et al. 2001a). Recent findings on over 100 tested cranial dura mater samples revealed an elastic modulus of 70 MPa (Zwirner et al. 2019c), which is more than twice as high as the aforementioned one. If unrealistic biomechanical properties are used in computational models, the predictions that are made based on these models such as the development of subdural bleedings related to particular head impact directions (Kleiven 2003) or the responses of the brain–spinal cord complex to particular head impacts (Kimpara et al. 2006) are likely invalid.

This given systematic review intends to summarize the reported biomechanical properties of the human cranial dura mater to date. The biomechanical properties of the spinal dura mater will also be systematically reviewed to analyze whether the same set of biomechanical properties can be used for both aspects of the dura mater. A meta-analysis will be performed that intends to establish the most appropriate elastic modulus value of the human dura mater to be used as a basis for graft developments, fundamental biomechanical research, and computational human head models. Factors that could have potentially influenced the mechanical properties reported in the studies such as sex of the donor, age of the donor at death, the brain region the sample was retrieved from or the presence of vessels at the tested samples will be considered. In addition, factors that are known to influence the biomechanical properties of the reported values such as testing speed (Saunders 2015) or the predominant collagen orientation within the tested sample (Runza et al. 1999; Zarzur 1996) will also be recorded.

## Materials and methods

### Study selection

A systematic literature review of peer-reviewed articles published up until June 2021 was performed according to the Preferred Reporting Items for Systematic Reviews and Meta-analyses (PRISMA) guidelines (Moher et al. 2010). The following online databases were used: Amed (1985 <), Embase (1947 <), and Medline (1949 <) via Ovid, also PubMed, ScienceDirect, Scopus and Web of Science (all approximately 1900 <). The following search combination was entered: “biomechanical AND (propert* OR parameter*) AND dura OR (dura AND mater)”. Microsoft Excel (Version 16.50; Microsoft Corporation, Redmond, WA, USA) was used to summarize the data and remove duplicates and non-primary research articles. Two authors (Q.P. and J.Z.) screened the titles and abstracts, then the full texts. In case of disagreement, a third reviewer (M.Z.) independently reviewed the study. All screened studies that contained information on the elastic modulus and/or ultimate tensile strength (UTS) and/or strain at maximum force were reviewed in their full text. Following the principle of the snowball search method, the reference list of included studies was screened in the same manner as studies emerging from the search of the databases. Only peer-reviewed studies in the English language using cadaveric tissues were included. The following exclusion criteria were defined: animal studies, non-peer-reviewed studies, non-primary research studies, and studies in languages other than English.

### Extraction of data

Data that fit the following categories were extracted from the selected studies: (i) demographic data of the studied cohort, (ii) information related to the mechanically tested samples, (iii) specifications regarding the used mechanical testing setup, and (iv) results of the mechanical tests. The demographic data included the number of different cadavers that were used in the experiments, the mean age of the cadavers at death (including standard deviation), the number of males and females in the tested cohort, and the post-mortem interval, which refers to the time between death of the cadaver and fixation or mechanical testing of the retrieved tissues. Information related to the mechanically tested samples included the number of tested samples, whether the mechanically tested dura mater samples originated from the cranium or the spine, whether the sample originated from a vascular or an avascular area of the dura mater, how the sample was cut with regard to the predominant collagen orientation that was macroscopically visible on the surface of the sample, the underlying brain region the sample was retrieved from, and the way the sample was stored and treated before the mechanical test. Specifications of the mechanical testing setup were recorded, including the testing speed of the mechanical tests (a speed of 120 mm/min was defined as the cutoff between static and dynamic), what medium the sample was tested in, the environmental temperature during the test and whether an optical analysis, such as Digital Image Correlation, was used to evaluate the biomechanical parameters. The results of the mechanical tests, the elastic modulus, the UTS, and the strain at maximum force were extracted from the studies.

### Meta-analysis

Meta-analysis was performed to determine the pooled mean estimate (PME) for the elastic modulus, UTS, and strain at maximum force of the dura mater. The collective values were studied and further analysis was performed when possible on the following subgroups: (i) cranial and all tissue preservation types, (ii) fresh cranial tissue only including all tissue testing conditions, (iii) fresh cranial tissue tested in air at room temperature, and (iv) fresh cranial tissue tested in a solution at 37 °C. Analysis of the effect size was determined using a random-effects model computed using the Comprehensive Meta-Analysis software (Version 3, Borenstein, M., Hedges, L., Higgins, J., & Rothstein, H., Biostat Inc., NJ, USA). The random-effects model was selected as information from cadaveric samples generally have high heterogeneity (Henry et al. 2016). By applying a random-effects model, this assumes a normal distribution within the sample that the effects are estimated, and different studies are not identical (Higgins et al. 2019). The PME analysis was reported with the 95% confidence intervals (CI), in order to address the issues of variance instability. Several inclusion criteria were required for the PME to be assessed or for a study to be included in the analyses: (i) studies with over 3 samples, (ii) studies that reported the sample size, mean and standard deviation, or for which this could be extrapolated from the results reported, (iii) prospective studies, and (iv) a minimum of 3 study groups investigating the same parameters. More than 10 studies on the topic were regarded as a substantial number to form valid conclusions from the literature. Retrospective studies were excluded as there is a potential risk of selection bias. The variance between studies included in the meta-analysis was studied using the *I*^2^ statistic, which assesses the amount of heterogeneity between studies. The variance was assessed using the following standard percentages: < 40% indicates low heterogeneity, 30–60% suggests moderate, 50–90% implies substantial, and 75–100% may be considerable heterogeneity (Higgins et al. 2019).

## Results

**Fig. 1:**
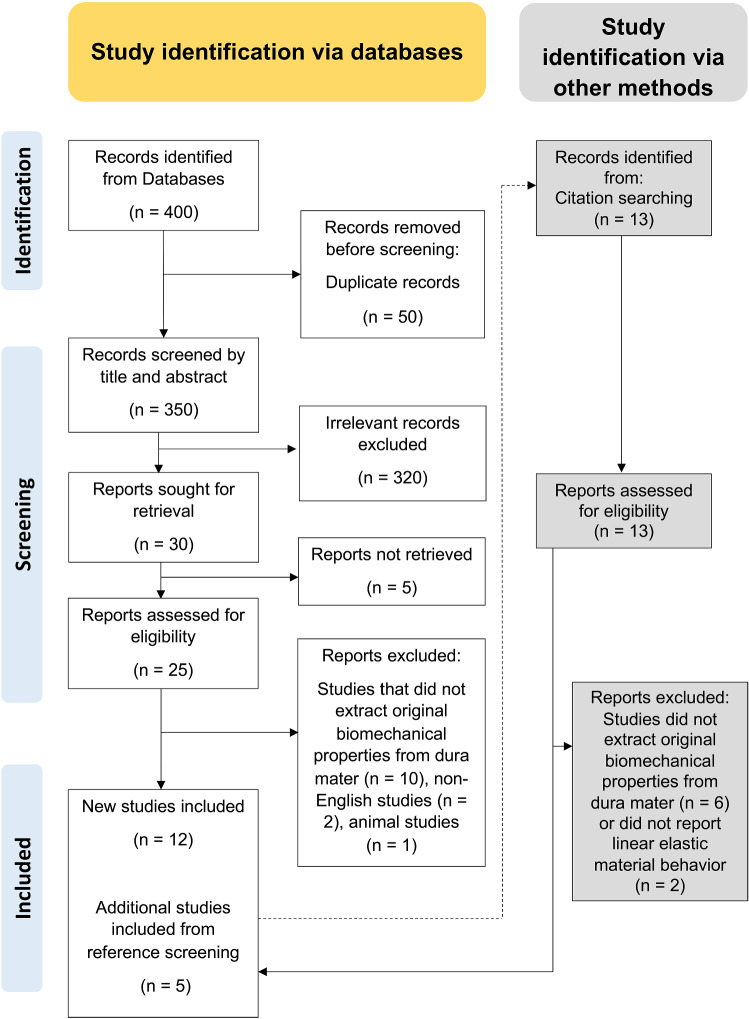
PRISMA flow chart for the methodology undertaken for the screening of relevant literature based on Moher et al. (2010)

A total of 17 papers were deemed eligible for inclusion from 400 records overall which were identified through databases and registers (see Fig. 1). These were published between 1970 and 2020. Of these, 13 studies were conducted on cranial (Aydin et al. 2019; Galford and McElhaney 1970; Kizmazoglu et al. 2019; McGarvey et al. 1984; Melvin et al. 1970; Sacks et al. 1998; van Noort et al. 1981; Wolfinbarger et al. 1994; Yamada et al. 1997; Zwirner et al. 2019a, b, c, 2020) and 4 on spinal dura mater (Patin et al. 1993; Runza et al. 1999; Tencer et al. 1985; Zarzur 1996), respectively. A summary of the retrieved information from the searched studies is given in Table 1.

**Table 1:** The table summarizes the information retrieved from previous studies on the biomechanical properties of the human dura mater

Authors	Sample/cadaver number	Cranial/spinal	Mean age (range) [years]	Females:males	Left-to-right ratio	PMI (range) [hours]	Sample storage and treatment	Test in fluid or air and temperature	Retrieval region of tested samples	Gauge length × width	Optical data analysis	Vascular/avascular areas of dura mater	Testing speed [mm/min]	Sample orientation	Assumption whether dura is isotropic/anisotropic	Testing mode	E_mod_ [MPa]	UTS [MPa]	SF_max_ [%]
**(**Zwirner et al. 2020**)**	124/75	Cranial	50 ± 24 (3 weeks to 94 years)	26:49	44:80	71 ± 31 Range 11 to 146	Fresh, stored at − 80 °C, gradually defrosted, water content adjusted over 24 h at 4 °C	Air (22 °C)	Temporal	10 × 5	N	Avascular	20	Macroscopically visible collagen bundles of surface layer orientated along load application axis in shaft area	Anisotropic	UTT	50 ± 22^a^	6 ± 4^a^	17 ± 4^a^
**(**Zwirner et al. 2019c**)**	117/73	Cranial	50 (2–94)	25:48	58:59	74 ± 30 Range 11–139	Fresh, stored at − 80 °C, gradually defrosted, water content adjusted over 24 h at 4 °C	Air (“room temperature”)	Temporal	10 × 5	Y	Avascular	20	Longitudinal^a^	Anisotropic	UTT	70 ± 44	7 ± 4	11 ± 3
**(**Kizmazoglu et al. 2019**)**	10/10	Cranial	43 ± 9 (31–54)	6:4	0:10	NS	Fresh, frozen at − 4 °C for 24–120 h before testing, thawed 6 h before testing	Artificial cerebrospinal fluid (37 °C)	Frontal	20 × 8	N	NS^b^	10	NS	Isotropic	UTT	60 ± 11	7 ± 1	NS
**(**Zwirner et al. 2019b**)**	12/6	Cranial	82 ± 8	3:3	6:6	NS	Thiel-embalmed	Air (22 °C)	Temporal	10 × 5	Y	Avascular	20	Longitudinal^a^	Anisotropic^a^	UTT	118 ± 68	9 ± 5	9 ± 2
	12/8^a^	Cranial	81 ± 8	3:5^a^	6:6	NS	Fresh, stored at − 80 °C, gradually defrosted, water content adjusted over 24 h at 4 °C	Air (22 °C)	Temporal	10 × 5	Y	Avascular	20	Longitudinal^a^	Anisotropic^a^	UTT	60 ± 14	6 ± 1	11 ± 2
**(**Aydin et al. 2019**)**	7/7	Cranial	45 ± 12 (31–62)	4:3	NS^c^	NS	Fresh, stored at − 20 °C, thawed at 4 °C for 24 h and 20–25 for 6 h	Saline solution (37 °C)	Frontal	20 × 8	N	NS^b^	10	NS	Isotropic	UTT	78 ± 41	8 ± 3	NS
**(**Zwirner et al. 2019a**)**	18/18	Cranial	48 (12–83)	6:12	10:8^a^	71 ± 28 (14–121)	Acellularized with sodiumdodecylsulfate	Air (“room temperature”)	Temporal	10 × 5	Y	Avascular	20	Longitudinal^a^	Anisotropic^a^	UTT	36 ± 12	4 ± 1	13 ± 2
	18/18	Cranial	48 (12–83)	6:12	10:8^a^	71 ± 28 (14–121)	Fresh, stored at − 80 °C, gradually defrosted, water content adjusted over 24 h at 4 °C	Air (“room temperature”)	Temporal	10 × 5	Y	Avascular	20	Longitudinal^a^	Anisotropic^a^	UTT	74 ± 26	7 ± 2	11 ± 2
**(**Runza et al. 1999**)**	6/6	Spinal	59 (39–86)	3:3	NS	NS	Fresh, in regular saline for less than 2 h	Air with 60% relative humidity (20 °C)	Dorsal lumbar (T-12-L5)	20 × 4	N	NS	10	Longitudinal	NS	UTT	83^d^	15^d^	41^d^
	6/6	Spinal	59 (39–86)	3:3	NS	NS	Dry^e^	Air with 60% relative humidity (20 °C)	Dorsal lumbar (T-12-L5)	20 × 4	N	NS	10	Longitudinal	NS	UTT	39^d^	7^d^	36^d^
	6/6	Spinal	59 (39–86)	3:3	NS	NS	Frozen for 24 h at − 4 °C	Air with 60% relative humidity (20 °C)	Dorsal lumbar (T-12-L5)	20 × 4	N	NS	10	Longitudinal	NS	UTT	82^d^	14^d^	27^d^
	6/6	Spinal	59 (39–86)	3:3	NS	NS	Frozen for 120 h at − 4 °C	Air with 60% relative humidity (20 °C)	Dorsal lumbar (T-12-L5)	20 × 4	N	NS	10	Longitudinal	NS	UTT	94^d^	14^d^	30^d^
	6/6	Spinal	59 (39–86)	3:3	NS	NS	Fresh, in regular saline for less than 2 h	Air with 60% relative humidity (20 °C)	Dorsal lumbar (T-12-L5)	20 × 4	N	NS	10	Perpendicular to longitudinal	NS	UTT	5^d^	4^d^	48^d^
**(**Sacks et al. 1998**)**	11/5	Cranial	54 ± 22	NS	NS	NS	Fresh, placed in saline then frozen with liquid nitrogen	Saline solution (“room temperature”)	NS	10 × 2^f^	Y	NS	10	Longitudinal	Anisotropic	UTT	193 ± 24	13 ± 2	13 ± 1
	12/5	Cranial	54 ± 22	NS	NS	NS	Fresh, placed in saline then frozen with liquid nitrogen	Saline solution (“room temperature”)	NS	10 × 2^f^	Y	NS	10	Perpendicular to longitudinal	Anisotropic	UTT	73 ± 11	5 ± 1	16 ± 2
**(**Zarzur 1996**)**	3/3	Spinal	56 ± 19 (38–73)	0:3	NS	NS	Preserved in formalin for 72 h	NS	Dorsal lumbar	20 × 20^f^	N	NS	20	Perpendicular to longitudinal	NS	UTT	19 ± 9	NS	45 ± 12
	3/3	Spinal	56 ± 19 (38–73)	0:3	NS	NS	Preserved in formalin for 72 h	NS	Dorsal lumbar	20 × 20^f^	N	NS	20	Longitudinal	NS	UTT	120 ± 45	NS	40 ± 12
**(**Wolfinbarger et al. 1994**)**	95/8	Cranial	40 ± 4 (17–51)	1:7	NS	NS	Fresh, freeze-dried; rehydrated under vacuum in physiological saline solution for 1 h	“Room temperature under consistent high humidity” (stored “on ice” for up to 3 h prior to testing)	“randomly cut” from entire dura samples	50 × 10^f^	N	NS	8	Longitudinal and perpendicular to longitudinal were included in the same evaluation	Isotropic	UTT	70 ± 4	7 ± 0	0.2 ± 0
**(**Patin et al. 1993**)**	7/7	Spinal	34 ± 25 (15 days to 62 years)	3:4	NA	NS	Fresh	NS	Dorsal lumbar	15 × 10^f^	N	NS	100	Longitudinal	NS	UTT	NS	81 ± 22	NS
	7/7	Spinal	34 ± 25 (15 days to 62 years)	3:4	NA	NS	Fresh	NS	Dorsal lumbar	15 × 10^f^	N	NS	100	Perpendicular to longitudinal	NS	UTT	NS	15 ± 22	NS
**(**McGarvey et al. 1984**)**	28/13	Cranial	52 (17–72)	3:10	NS	NS	Fresh, stored in Hanks balanced salt solution, tested within 20 h of autopsy	Tested in Hanks balanced salt solution (37 °C)	NS	10 × 7^f^	N	NS	10	Longitudinal and perpendicular to longitudinal were grouped in evaluation	Isotropic	UTT	62 ± 10	9 ± 2	32 ± 2
	26/13	Cranial	52 (17–72)	3:10	NS	NS	98% glycerol 13 days–7 weeks	Tested in Hanks solution (37 °C)	NS	10 × 7^f^	N	NS	10	Longitudinal and perpendicular to longitudinal were grouped in evaluation	Isotropic	UTT	45 ± 3	6 ± 1	25 ± 1
**(**van Noort et al. 1981**)**	12^f^	Cranial	20–77	NS (“from both male and female cadavers”)	NS	Up to 12	Fresh, put into saline solution for up to 5 h	NS	NS	40 × 5^f^	N	Avascular	50	“No particular orientation was chosen”	Isotropic	UTT	29 ± 8^g^	5 ± 1^g^	18 ± 1^g^
	12^f^	Cranial	20–77	NS (“from both male and female cadavers”)	NS	Up to 12	Put into saline solution for up to 5 h, 98% glycerol for 1–12 days	NS	NS	40 × 5^f^	N	Avascular	50	“No particular orientation was chosen”	Isotropic	UTT	30 ± 8^g^	5 ± 1^g^	18 ± 2^g^
**(**Tencer et al. 1985**)**	5 (cadaver number not specified)	Spinal	Up to 65	NS	NA	NS	Fresh, stored at − 20 °C	NS	Cervical, low and high thoracic, anterior and posterior lumbar region	38.1 × 2.5	N	NS	NS	Longitudinal	NS	UTT	151^h^	28	34
**(**Melvin et al. 1970**)**	Over 100 (cadaver number not specified)^i^	Cranial	NS	NS	NS	NS	Fresh, refrigerated in saline solution if not tested immediately	NS	NS	19.1 × 6.4	N	“Relatively free from large blood vessels”	2.28	Longitudinal, perpendicular to longitudinal and diagonal (included in evaluation together)	NS	UTT	48^j^	NS	NS
**(**Yamada et al. 1997**)**	15/15	Cranial	31 ± 21 (3–62)	NS	NS	NS	NS	Sprayed with normal saline during test (“room temperature”)	NS	10 × 5^f^	N	NS	50	NS	NS	UTT	3 ± 1	9 ± 2	NS
**(**Galford and McElhaney 1970**)**	11/2	Cranial	NS	NS	NS	6–12	Fresh, kept moist (fluid not explicitly specified)	Kept moist (fluid not explicitly specified)	NS	2.5 × 6.4^f^	N	NS	NS	NS	NS	Tensile-free vibration test (21 Hz)	31.5	NS	NS

*NS* not stated, *UTT* ultimate tensile test

**Fig. 2:**
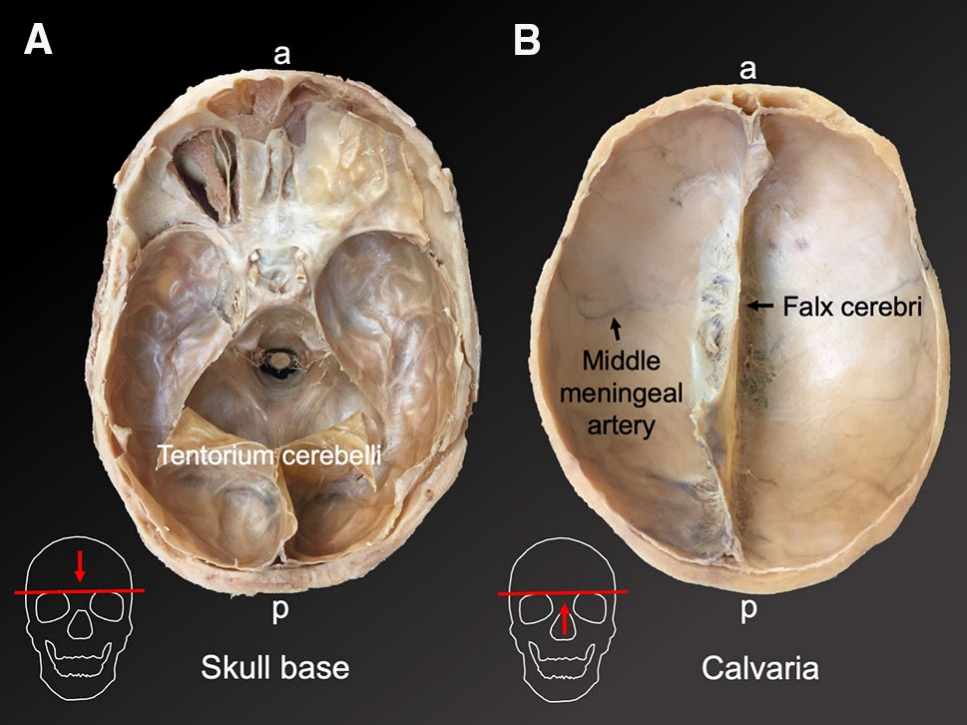
The human cranial dura mater is firmly attached to the inner aspect of the neurocranium (depicted on plastinates of the W D Trotter Museum of the University of Otago, Dunedin, New Zealand). **A** The skull base is covered with dura mater (apart from the left anterior skull base, where the dura mater and the roof of the orbit were removed). The tentorium cerebelli represents a dura mater extension that separates the cerebellum from the occipital lobe. **B** Remarkable dura mater structures of the calvaria are the falx cerebri and the meningeal vessels. *a* anterior, *p* posterior

^a^Information provided by the authors upon request; ^b^Vessels present in manuscript figures; ^c^Stated that both left and right samples were used; ^d^Values were calculated by averaging the minima and maxima that were read from Fig. 2; ^e^Not specified how samples were dried; ^f^“Gauge” not explicitly stated, not stated that samples were cut into dumbbell shape, values indicate grip-to-grip length; ^g^Averaged from data presented in this Table; ^h^Average of cervical and lumbar value; ^i^Up to 11 samples were cut per dura mater; ^j^Average of range presented in study

### Studies on fresh human cranial dura mater

The sample characteristics varied between studies. The number of mechanically investigated samples ranged from 7 (Aydin et al. 2019) to 124 (Zwirner et al. 2020). Cadaver numbers ranged from 5 (Sacks et al. 1998) to 75 (Zwirner et al. 2020). In one case, the difference between tested samples and number of investigated cadavers was not stated clearly (Melvin et al. 1970). Three weeks to 94 years was the largest investigated age span both within a single study and across all studies (Zwirner et al. 2020). The mean age at death for fresh cranial dura mater samples across all studies that reported this information was 49 ± 13 years, this was calculated by averaging the reported mean ages within the studies. The overall female to male ratio was 1:1.9 (Aydin et al. 2019; Galford and McElhaney 1970; Kizmazoglu et al. 2019; McGarvey et al. 1984; Melvin et al. 1970; Sacks et al. 1998; van Noort et al. 1981; Wolfinbarger et al. 1994; Yamada et al. 1997; Zwirner et al. 2019a, b, c, 2020); however, 5 of the 13 studies on human cranial dura mater did not report the sex ratio of the tested samples (Galford and McElhaney 1970; Melvin et al. 1970; Sacks et al. 1998; van Noort et al. 1981; Yamada et al. 1997). The overall left-to-right ratio was 1:1.4, which was reported by only 5 studies (Kizmazoglu et al. 2019; Zwirner et al. 2019a, b, c, 2020). Only 3 studies reported a precise post-mortem interval, which averaged 72 h with a span between 11 and 146 h (Zwirner et al. 2019a, c, 2020). In one group, it was mentioned that the samples were tested within 12 h after death but no precise average was provided (van Noort et al. 1981).

The tested samples were retrieved from the frontal (Aydin et al. 2019; Kizmazoglu et al. 2019) and temporal cranial regions (Zwirner et al. 2019a, b, c, 2020) or were “randomly cut” from the entire dura mater (Wolfinbarger et al. 1994) (see Fig. 2). Anatomical detail on the specific sampling site was lacking in 6 studies (Galford and McElhaney 1970; McGarvey et al. 1984; Melvin et al. 1970; Sacks et al. 1998; van Noort et al. 1981; Yamada et al. 1997). Six studies in total stated that the samples for biomechanical testing were taken from avascular (van Noort et al. 1981; Zwirner et al. 2019a, b, c, 2020) or areas that were “relatively free from large blood vessels” (Melvin et al. 1970). The remaining studies did not specify whether vascular areas of the dura mater were included; however, in Fig. 1 of the study of Kizmazoglu et al. (2019), vessels are clearly present in the tested sample. The anatomical location of the human cranial dura mater is depicted in Fig. 2.

Between retrieval and biomechanical testing, researchers made efforts to keep the samples moist and prevent tissue deterioration. To prevent sample dehydration, the tissues were kept in saline solution (Melvin et al. 1970; van Noort et al. 1981), Hanks balanced salt solution (McGarvey et al. 1984) or moist using an unspecified liquid (Galford and McElhaney 1970) until mechanical testing. Other groups froze the samples between retrieval and testing at − 4 °C (Kizmazoglu et al. 2019), − 20 °C (Aydin et al. 2019), − 80 °C (Zwirner et al. 2019a, b, c, 2020) or freeze-dried them (Wolfinbarger et al. 1994). Others placed them in saline and then froze them with liquid nitrogen (Sacks et al. 1998). One group did not report how the samples were stored between retrieval mechanical testing (Yamada et al. 1997). Mechanical testing was performed either in fluid (Aydin et al. 2019; Kizmazoglu et al. 2019; McGarvey et al. 1984; Sacks et al. 1998) or in air (Galford and McElhaney 1970; Wolfinbarger et al. 1994; Zwirner et al. 2019a, b, c, 2020). When tested in air, it was reported that “consistent high humidity” was assured (Wolfinbarger et al. 1994), samples were “sprayed with normal saline solution” (Yamada et al. 1997), “kept moist” (Galford and McElhaney 1970) or sample hydration was assured using the osmotic stress technique prior to testing with minimal time intervals in air of about two to three minutes until the mechanical tests were finished (Zwirner et al. 2019a, b, c, 2020). However, the testing environment was insufficiently reported in some studies (Melvin et al. 1970; van Noort et al. 1981).

**Fig. 3:**
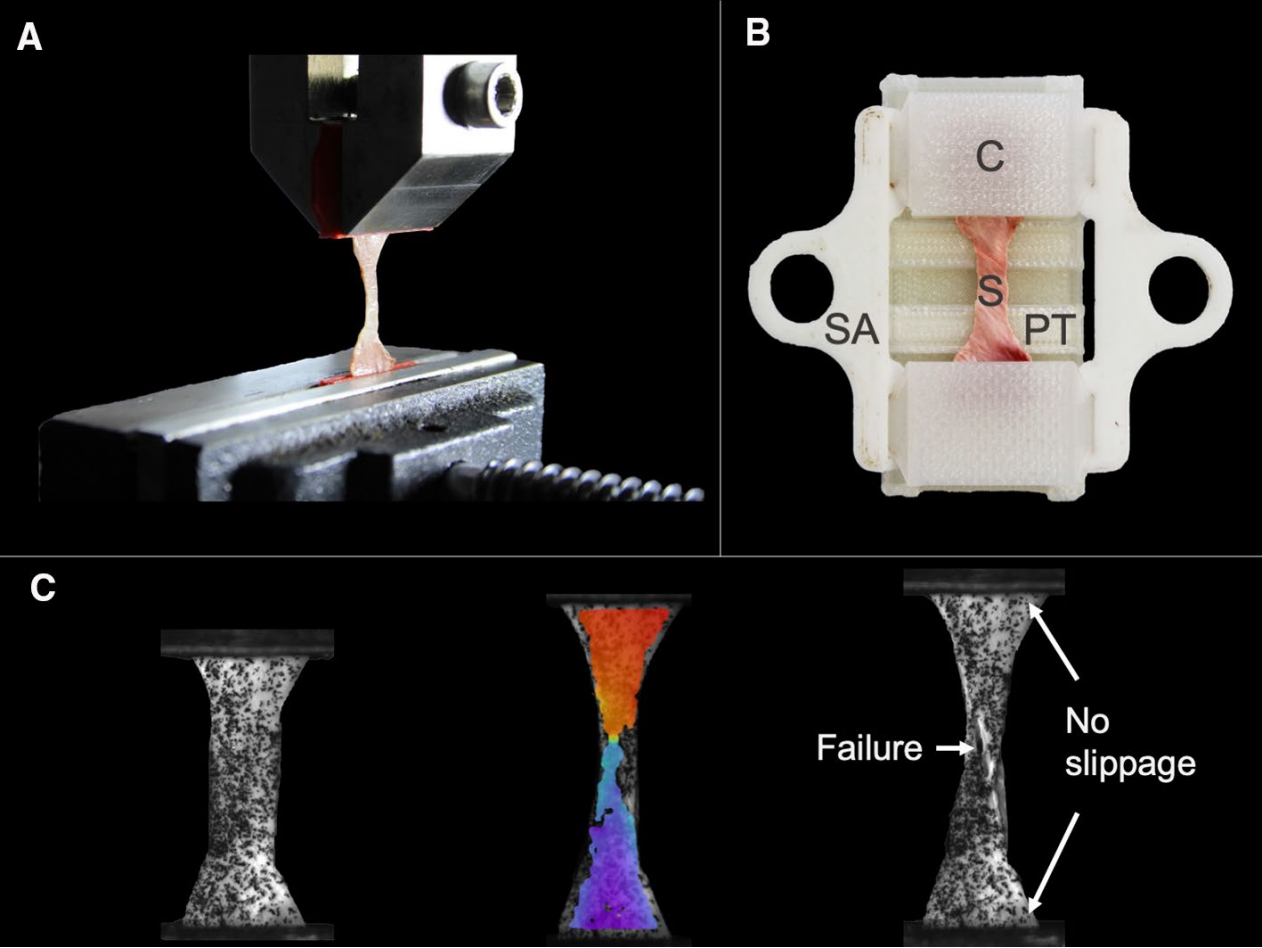
Illustrations of human cranial dura mater testing from the senior author’s lab are depicted. **A** An uniaxial tensile testing setup is shown, which is the most commonly used setup for dura mater tests in the literature. **B** 3D-printed equipment that assured fast and consistent sample handling was used by several studies in the literature (Zwirner et al. 2019a, b, c, 2020). **C** An optical data evaluation, which was performed by several studies in the literature (Sacks et al. 1998; Zwirner et al. 2019a, b, c), allows verifying the failure point and detecting potential specimen slippage during the mechanical test. *C* clamp, *S* sample, *SA* supporting arms, *PT* preparation table

**Fig. 4:**
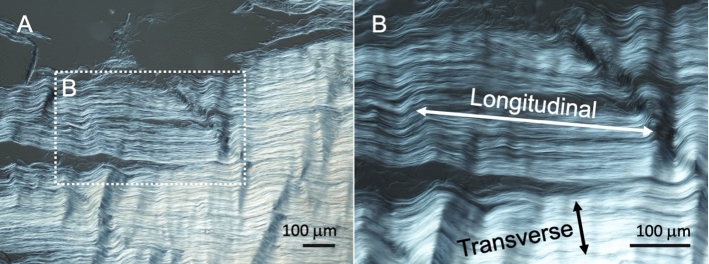
Differential interference contrast microscope images of the human cranial dura mater are depicted. **A** Highly aligned collagen bundles are observed. **B** So far, the cranial dura mater was mostly tested longitudinally (along the collagen bundle axis) rather than transversely (perpendicular to the preferred course)

The study by Galford and McElhaney (1970) was the only one to use a dynamic testing setup rather than a quasi-static one to determine the linear elastic properties of human cranial dura mater. Apart from one tensile-free vibration test (Galford and McElhaney 1970), ultimate tensile tests have been the chosen setup for the determination of the biomechanical properties in all cases (Aydin et al. 2019; Galford and McElhaney 1970; Kizmazoglu et al. 2019; McGarvey et al. 1984; Melvin et al. 1970; Sacks et al. 1998; van Noort et al. 1981; Wolfinbarger et al. 1994; Yamada et al. 1997; Zwirner et al. 2019a, b, c, 2020) (see Fig. 3A). Optical evaluation of the biomechanical parameters that aids in controlling for sample slippage during the test was performed in four studies (Sacks et al. 1998; Zwirner et al. 2019a, b, c) (see Fig. 3C). With regard to the macroscopically visible preferred collagen orientation of the sample, studies reported that they tested the samples longitudinal (Zwirner et al. 2019a, b, c, 2020), longitudinal and transverse reporting the two groups separated (Sacks et al. 1998), longitudinal and transverse pooled in one evaluation (McGarvey et al. 1984; Wolfinbarger et al. 1994), longitudinal, transverse, and diagonal pooled in one evaluation (Melvin et al. 1970), or without respecting any particular orientation (van Noort et al. 1981) (see Fig. 4). The orientation was not specified in four of the studies (Aydin et al. 2019; Galford and McElhaney 1970; Kizmazoglu et al. 2019; Yamada et al. 1997).

### Studies on chemically altered cranial human dura mater

In four studies, the biomechanical properties of human cranial dura mater were investigated in which the samples were chemically treated between retrieval and mechanical testing (McGarvey et al. 1984; van Noort et al. 1981; Zwirner et al. 2019a, b) using embalming solution and solutions to restore the water content of the tissue. The elastic modulus of 118 ± 68 MPa of Thiel-embalmed human cranial dura mater was significantly higher compared to values produced by fresh tissue with 60 ± 14 MPa (*p* < 0.01) (Zwirner et al. 2019b). A comparison of native and acellular cranial dura mater samples showed that the presence of cells seems to be negligible for the elastic modulus, UTS, or strain at maximum force (Zwirner et al. 2019a). Glycerol treatment of up to 12 days led to an increase of the UTS but showed statistically non-different values for the elastic modulus and the strain at maximum force (van Noort et al. 1981). Treatment with 98% pure glycerol between 13 days and 7 weeks led to an increased elastic modulus at strains below 1.6 MN/m^2^ but was statistically non-different at higher strains when compared to fresh samples (McGarvey et al. 1984). Both UTS and maximum strain were significantly lower when compared to fresh cranial dura mater samples (McGarvey et al. 1984).

### Studies on fresh human spinal dura mater

The number of mechanically characterized fresh spinal dura mater samples between the studies ranged from 3 (Zarzur 1996) to 7 (Patin et al. 1993) with all of those being from different cadavers. One study did not specify the number of investigated cadavers (Tencer et al. 1985). The average age at death of the investigated cadaveric samples was 47 years with an age span of 15 days to 86 years (Patin et al. 1993; Runza et al. 1999). Another study only reported that the mechanically tested samples were taken from cadavers “up to 65 years” at death (Tencer et al. 1985). Including only the studies that reported it, the female-to-male ratio was 6:7 (Patin et al. 1993; Runza et al. 1999). No post-mortem interval was specified for the mechanically tested fresh human dura mater samples (Patin et al. 1993; Runza et al. 1999; Tencer et al. 1985).

With regard to storage between retrieval and testing, one study investigated different groups including fresh samples stored in saline for two hours and samples that were frozen for 24 and 120 h at − 4 °C, respectively (Runza et al. 1999). The testing environment was only specified in one study, which was performed in air with 60% relative humidity at 20 °C (Runza et al. 1999). The retrieval site of the spinal dura mater was dorsal lumbar (Patin et al. 1993; Runza et al. 1999; Tencer et al. 1985) and additionally anterior lumbar, low and high thoracic and cervical in one study (Tencer et al. 1985). However, only results of the cervical and lumbar dura mater were presented in the latter (Tencer et al. 1985). The biomechanical properties of fresh lumbar dura mater samples were determined based on the values gained from the testing machines rather than optically using, e.g., Digital Image Correlation (Patin et al. 1993; Runza et al. 1999; Tencer et al. 1985). It was not mentioned so far, if macroscopically visible vessels were part of the biomechanically tested spinal dura mater samples. Testing velocities were quasi-static with velocities ranging between 10 mm/min (Runza et al. 1999) and 100 mm/min (Patin et al. 1993); however, it was not specified in one study (Tencer et al. 1985). All studies were performed using ultimate tensile tests (Patin et al. 1993; Runza et al. 1999; Tencer et al. 1985). The samples were taken longitudinally (Patin et al. 1993; Runza et al. 1999; Tencer et al. 1985) and perpendicular to longitudinally (Runza et al. 1999) with respect to the underlying collagen bundles of the spinal dura mater.

### Studies on chemically altered human spinal dura mater

One study reported the biomechanical properties of the human spinal dura mater, which was submerged in formalin for 3 days (Zarzur 1996). Three longitudinal samples of the dorsal lumbar region of the spine were compared to transverse samples of the same three male cadavers (Zarzur 1996). A statement of whether vessels were present in the tested samples was absent (Zarzur 1996). The average age at death of the used cadavers was 56 ± 19 years with an age span of 38 to 73 years (Zarzur 1996). The post-mortem interval, referring to the time between harvesting and preserving the samples in formalin in this case, was not specifically mentioned (Zarzur 1996). The samples were tested in a quasi-static ultimate tensile testing setup without the application of Digital Image Correlation (Zarzur 1996). The results of the study revealed that the elastic modulus of longitudinal samples with 120 MPa was significantly higher compared to the elastic modulus of transverse samples, which averaged 19 MPa (*p* = 0.05) (Zarzur 1996). Moreover, longitudinal samples withstood significantly higher loads with 50 MPa compared to 10 MPa of transverse samples (*p* = 0.05) (Zarzur 1996).

### Meta-analysis

**Fig. 5:**
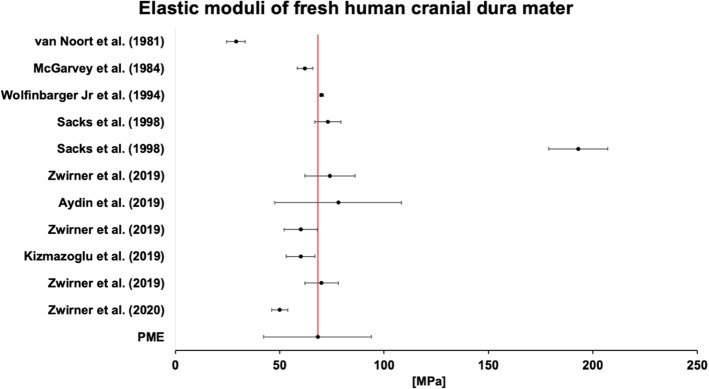
Individual study results and the pooled mean estimate of the linear elastic moduli of fresh human cranial dura mater samples are depicted

A maximum of 18 of the 28 study groups found in this systematic literature search were able to be included in the meta-analysis (see Table 2). The most common reason for exclusion was the limited information available in these studies, particularly the absence of a standard deviation value. The sample size for meta-analysis ranged from 45 to 600 samples included in the estimates. All *p* values were significant. The heterogeneity of studies included in each meta-analysis was variable. All calculated PME values for the elastic modulus and strain at maximum force had low heterogeneity. When further examining the reliability of the UTS values, the *I*^2^ statistic for pooled results showed considerable heterogeneity, whereas the values for "all cranial" and "cranial and fresh" were moderately homogeneous and had a small degree of standard error. This heterogeneity decreased further when separating the data according to the testing conditions. Values reported for UTS of native cranial dura mater tested in air at room temperature were substantially different to those tested in a solution at 37 °C. The biomechanical parameters for the spinal dura mater could not be reported for the elastic modulus, UTS, or strain at maximum force due to the small number of studies investigating this tissue, nor could the strain at maximum force of cranial samples tested at 37 °C in a solution, as this had not been performed to date. The elastic moduli of fresh human cranial dura mater are summarized in Fig. 5.

**Table 2:** The meta-analysis results are depicted

	Tissue group	Samples	Groups	PME	Standard error	Confidence interval (95%)		*I*^2^	*p* value
						Lower limit	Upper limit		
E_mod_	All*	535	18	64.3	9.4	45.9	82.6	15.5	0.000
	Cranial only*	529	16	64.6	9.9	45.3	84.0	13.3	0.000
	Cranial only (fresh)	446	11	68.1	13.1	42.3	94.0	0	0.000
	Cranial, RT, air (fresh)	271	4	62.8	6.0	51.1	74.5	0	0.000
	Cranial, 37 °C, solution (fresh)	45	3	61.7	1.7	58.5	65.0	0	0.000
UTS	All*	549	18	7.1	0.4	6.3	7.8	84.4	0.000
	Cranial only*	529	16	6.9	0.4	6.2	7.6	57.5	0.000
	Cranial only (fresh)	446	11	7.2	0.4	6.4	8.1	58.2	0.000
	Cranial, RT, air (fresh)	271	4	6.4	0.3	5.9	7.0	0.3	0.000
	Cranial, 37 °C, solution (fresh)	45	3	8.0	0.8	6.4	9.6	0	0.000
SF_max_	All	503	15	18.0	3.6	11.0	25.0	0	0.000
	Cranial only*	497	13	15.0	3.8	7.6	22.3	0	0.000
	Cranial (fresh)	429	9	14.4	4.0	6.4	22.3	0	0.000
	Cranial, RT, air (fresh)	271	4	12.5	1.6	9.3	15.7	0	0.000

*E*_*mod*_ elastic modulus, *RT* room temperature, *SF*_*max*_ strain at maximum force, *UTS* ultimate tensile strength, *PME* pooled mean estimate

*Including all forms of preservation between tissue retrieval and testing

## Discussion

### The simulation of the elastic behavior of the human cranial dura mater in computational head models should be revised

An elastic modulus of 31.5 MPa that was established in dynamic free vibration tests (Galford and McElhaney 1970) is the most commonly used value to simulate the human cranial dura mater in computational head models (Chafi et al. 2009; Kleiven 2003; Viano et al. 2005; Zhang et al. 2001a). This value is based on the comparatively low number of only eleven mechanical tests from a total of two different cadavers (Galford and McElhaney 1970). Moreover, the demographic and anatomical data of the tested samples were scarce preventing the study from being replicated (Galford and McElhaney 1970). A recent quasi-static study on the material properties of fresh cranial dura mater in a large sample size revealed a more than two times higher value for the elastic modulus of 70 MPa (Zwirner et al. 2019c). The comparison of these two values contradicts the fundamental biomechanical rule that the elastic modulus of biological tissues should increase with increasing strain rates (Saunders 2015). Alternative explanations could be that the specimens tested by Galford and McElhaney (1970) were retrieved from another brain region or were more hydrated at the time of testing (Lozano et al. 2019), which is difficult to comprehend given the little information shared by Galford and McElhaney (1970). To get clarity on what the most appropriate biomechanical properties are to simulate the cranial human dura mater in computational models, the here given systematic literature review including a meta-analysis was conducted. The pooled mean estimate for the elastic modulus of the 11 studies on fresh human cranial dura mater including a total of 448 tested samples was 68 ± 13 MPa (Aydin et al. 2019; Galford and McElhaney 1970; Kizmazoglu et al. 2019; McGarvey et al. 1984; Melvin et al. 1970; Sacks et al. 1998; van Noort et al. 1981; Wolfinbarger et al. 1994; Zwirner et al. 2019a, b, c, 2020). Apart from one quasi-static study that was conducted on 12 cadavers (van Noort et al. 1981), all values for the elastic modulus of fresh human cranial dura mater were higher than the single dynamic elastic modulus value provided by Galford and McElhaney (1970). Hence, it is likely that a dynamic elastic modulus value of 31.5 MPa is an underestimation and should, therefore, not be used to simulate the elasticity of the human cranial dura mater. The difference between the PMEs for the elastic modulus of dura mater samples that were tested at room temperature in air and at 37 °C in a solution were negligible. However, this remains to be experimentally confirmed in a future study.

Given that predominantly quasi-static testing velocities were used to determine the biomechanical properties of the human cranial dura mater, current values may not be representative of the forces applied to the dura during head impacts, such as sustained in falls, gunshots, or contacts sports, which are likely of a dynamic nature (Brooks et al. 2021). Therefore, there is an urgent need to explore the dynamic biomechanical properties of the human cranial dura mater in future studies. Only this will allow simulating the cranial dura mater appropriately in computational head models, which enhance the quality of the conclusions that are drawn from such models.

### Accurate biomechanical properties of human cranial dura mater serve various applications

Accurate data on the mechanical strength of the human cranial dura mater are required to fabricate artificial substitutes that mimic the mechanical behavior of the original tissue (Kizmazoglu et al. 2019). The meta-analysis included in this study revealed an UTS value of 7.2 ± 0.4 MPa for fresh cranial dura mater, which is based on 446 tested samples from 11 studies (Aydin et al. 2019; Galford and McElhaney 1970; Kizmazoglu et al. 2019; McGarvey et al. 1984; Melvin et al. 1970; Sacks et al. 1998; van Noort et al. 1981; Wolfinbarger et al. 1994; Zwirner et al. 2019a, b, c, 2020). A precise knowledge of the fresh biomechanical properties of the human dura mater allows the biomechanical effect of different treatments of the tissue to be assessed. It is of practical interest to determine whether embalmed dura mater specimens can be used for biomechanical studies as fresh specimens are often unavailable to research labs. It was shown that the Thiel embalming had an insignificantly higher UTS of 9 MPa compared to fresh dura mater (Zwirner et al. 2019b). However, Thiel-embalmed samples were significantly stiffer compared to unembalmed tissues (Zwirner et al. 2019b). The increased stiffness of Thiel-embalmed dura mater samples was attributed to collagen crosslinking due to formaldehyde, which yet has to be confirmed experimentally (Zwirner et al. 2019b).

The meta-analysis demonstrates that distinct values are produced for UTS of cranial dura mater tested in air at room temperature (6.4 MPa) compared to those tested in a solution at 37 °C (8 MPa). This suggests that these variables likely have an effect on the UTS of cranial dura and should be assessed and considered in future testing setups. Recently, the cranial dura mater has been used as a model tissue to investigate the impact of cells on the biomechanical behavior of collagen-rich tissues (Zwirner et al. 2019a) or study influencing factors on the biomechanical properties of collagen-rich soft tissues that are obtained in tensile tests (Zwirner et al. 2020).

### Profound structural and mechanical differences between cranial and spinal dura mater necessitate a separate simulation in computational models

This systematic review revealed that the biomechanical properties of the human spinal dura mater are scarce (Patin et al. 1993; Runza et al. 1999; Tencer et al. 1985; Zarzur 1996) with seven different cadavers being the highest number investigated within a single study (Patin et al. 1993). Hence, it was impossible to perform a meta-analysis of the mechanical properties of the human spinal dura mater in this systematic review. Therefore, the question arises whether the biomechanical properties of cranial and spinal dura mater can be used interchangeably. Both cranial and spinal dura mater reveal aligned collagen bundles, which were respected in several studies when cutting the samples for mechanical testing (McGarvey et al. 1984; Patin et al. 1993; Runza et al. 1999; Sacks et al. 1998; Tencer et al. 1985; Wolfinbarger et al. 1994; Zarzur 1996; Zwirner et al. 2019a, b, c, 2020). Furthermore, a study conducted on the cranial and spinal dura mater in rats (Maikos et al. 2008) reported that the elastin content in the spinal dura mater seemed to be significantly higher compared to cranial dura mater. Therefore, structural differences between the two dura sites might explain the observed differences in mechanical behavior. These differences might even exist within the different spinal segments of the human dura mater, which has to be elucidated in future anatomical studies. Future studies should attempt to couple mechanical investigations with structural analyses to deepen the understanding of the structure–function relation of the human dura mater. While cranial dura samples were tested predominantly longitudinally (Zwirner et al. 2019a, b, c, 2020) or the results of longitudinal and transverse samples were pooled (McGarvey et al. 1984; Wolfinbarger et al. 1994), for spinal dura mater longitudinal and transverse samples were commonly reported independently (Patin et al. 1993; Runza et al. 1999; Zarzur 1996). Longitudinal samples of spinal dura mater were on average stiffer and stronger in uniaxial tensile tests when compared to transverse samples, hence indicating a transversely isotropic behavior of the dura mater (Patin et al. 1993; Runza et al. 1999; Zarzur 1996). Even though no statistical comparison between cranial and spinal dura mater is available, this systematic review revealed that longitudinal spinal samples seem to be stiffer and stronger when compared to cranial dura mater samples (Aydin et al. 2019; Galford and McElhaney 1970; Kizmazoglu et al. 2019; McGarvey et al. 1984; Melvin et al. 1970; Patin et al. 1993; Runza et al. 1999; Tencer et al. 1985; van Noort et al. 1981; Yamada et al. 1997; Zarzur 1996; Zwirner et al. 2019a, b, c, 2020). Only one study on 11 cranial dura mater samples (Sacks et al. 1998) observed an elastic modulus that was similar to the elastic modulus of longitudinal spinal samples (Patin et al. 1993; Runza et al. 1999; Tencer et al. 1985; Zarzur 1996). Transverse spinal samples (Patin et al. 1993; Runza et al. 1999; Zarzur 1996) seem to be more elastic compared to cranial dura samples (Aydin et al. 2019; Galford and McElhaney 1970; Kizmazoglu et al. 2019; McGarvey et al. 1984; Melvin et al. 1970; Sacks et al. 1998; van Noort et al. 1981; Wolfinbarger et al. 1994; Yamada et al. 1997; Zwirner et al. 2019a, b, c, 2020). Conclusively, it should be recommended to use the site-specific biomechanical properties of human dura mater when simulating the tissue in computational models.

### Considerations for future biomechanical studies on human dura mater

In this literature review, several gaps were identified that should be investigated in future mechanical characterizations of both the human cranial and spinal dura mater. The biomechanical properties together with microstructural analysis of the human dura mater should be explored more in-depth using dynamic and multiaxial testing setups. It is expected that the former is superior when the biomechanical behavior of the human dura mater is simulated in computational models that investigate dynamic impacts such as falls (Raul et al. 2006), gunshots (Raul et al. 2007) or traffic accidents (Yang et al. 2014). The influence of freeze-thaw cycles, storage conditions, and the testing environment (including the temperature, humidity and testing within a fluid) should be investigated further, and it is recommended that any study on the human dura mater reports these aspects in detail. A circumstantial reporting of the mechanically tested samples’ demographic data such as left-to-right ratio, female-to-male ratio, age at death of the cadavers, past medical history, cause of death, age span of the cadavers, and both the number of tested samples and cadavers should be mandatory to abide by the recommendations of the AQUA checklist (Tomaszewski et al. 2017). Analyzing this data will help to answer the question of whether the biomechanical properties of the human dura mater in computational head models have to be adjusted for anatomical region, age, sex, or bodyside. Furthermore, it should be explored whether vascular areas of the dura mater with embedded vessels such as the middle meningeal artery significantly differ from avascular areas of the dura mater. It is important to know whether the presence of vessels significantly impairs the strength of the dura mater, which then should be considered when it is used as a tissue graft.

This literature review highlighted the importance of reporting the collagen orientation of the tested samples. As a further anatomical aspect, the influence of dural sinuses, arachnoid granulations, and large vessels such as the middle meningeal artery on the biomechanics of the human dura mater should be explored in future studies. For cranial dura mater, efforts should be made that all brain regions are represented in the mechanical studies including the skull base. Specifically, for the spinal dura mater, future studies should include representative sections of the entire spine including the anterior dura mater and all different segments from spinal to lumbar.

## Limitations

This literature review focused on mechanical characterization of the human dura mater using a linear elastic modulus, UTS, and the strain at maximum force as the parameters of interest. This has been performed as the current simulation of the dura mater using the elastic modulus provided by Galford and McElhaney (1970) is based on this linear elastic model, which was deemed sufficient by the authors to describe the mechanical behavior of the dura mater. Other research groups argued that the human cranial dura mater is, in fact, a non-linear elastic material (Bylski et al. 1986; De Kegel et al. 2018) and proposed a simulation of the dura mater based on a Neo-Hookean formulation (De Kegel et al. 2018), which, however, assumes an isotropic material behavior. An isotropic material behavior is contradicted by the orientation-dependent elastic properties of the human cranial dura mater (Patin et al. 1993; Runza et al. 1999; Zarzur 1996). Therefore, a transversely isotropic model should be recommended for future mechanical characterizations of the human dura mater. For the study selection of this systematic literature review, it was agreed that with regard to computational efficiency and when analyzing head impacts where the dura mater is off the main focus, a linear elastic model would still be considered appropriate (De Kegel et al. 2018). However, this has to be thoroughly investigated and yet remains an open question. A linear isotropic elastic material model requires at least two constants such as the elastic modulus and Poisson’s ratio. Poisson’s ratio has not been in the focus of this review as, to the best of the author’s knowledge, no original data have been reported for the human dura mater to date. For finite element models of the cranial human dura mater, a Poisson’s ratio of 0.45 is most frequently used (Hu et al. 2007; Voo et al. 1996; Yan and Pangestu 2011; Zhang et al. 2001b) without referencing original data that support that value, an assumption based on the nearly incompressible nature of human soft tissues. Furthermore, some studies may have been missed due to the exclusion of non-English literature and non-peer-reviewed articles. Lastly, the here performed meta-analysis did not respect factors such as age, sex, post-mortem interval, or sample thickness as factors with a potential influence on the here synthesized biomechanical parameters.

## Conclusion

The most commonly used elastic modulus value of 31.5 MPa for the simulation of the human cranial dura mater in computational head models is likely an underestimation. Based on the meta-analysis results, an elastic modulus of 61.7 MPa was determined for native cranial dura mater. Future mechanical characterizations of the human dura mater should further investigate dynamic and multiaxial mechanical properties of the dura mater as well as non-linear material models. Correlations between the mechanical parameters and sample characteristics are paramount for an in-depth understanding of the dura mater tissue mechanics.

## Data Availability

Not applicable.
